# Botanical microbiomes on the cheap: Inexpensive molecular fingerprinting methods to study plant‐associated communities of bacteria and fungi

**DOI:** 10.1002/aps3.11334

**Published:** 2020-04-15

**Authors:** David Johnston‐Monje, Jessica Lopez Mejia

**Affiliations:** ^1^ Max Planck Tandem Group in Plant Microbial Ecology at the Universidad del Valle Calle 13 #100‐00, Building E20 760032 Cali, Valle del Cauca Colombia; ^2^ Max Planck Institute for Plant Breeding Research Department of Plant Microbe Interactions Carl-von-Linne-Weg 10 D-50829 Cologne Germany

**Keywords:** community fingerprinting, denaturing gradient gel electrophoresis (DGGE), endophytes, microbiome, mycobiome, phytobiome, rhizosphere, terminal restriction fragment length polymorphism (TRFLP)

## Abstract

High‐throughput sequencing technologies have revolutionized the study of plant‐associated microbial populations, but they are relatively expensive. Molecular fingerprinting techniques are more affordable, yet yield considerably less information about the microbial community. Does this mean they are no longer useful for plant microbiome research? In this paper, we review the past 10 years of studies on plant‐associated microbiomes using molecular fingerprinting methodologies, including single‐strand conformation polymorphism (SSCP), denaturing gradient gel electrophoresis (DGGE), amplicon length heterogeneity PCR (LH‐PCR), ribosomal intergenic spacer analysis (RISA) and automated ribosomal intergenic spacer analysis (ARISA), and terminal restriction fragment length polymorphism (TRFLP). We also present data juxtaposing results from TRFLP methods with those generated using Illumina sequencing in the comparison of rhizobacterial populations of Brazilian maize and fungal surveys in Canadian tomato roots. In both cases, the TRFLP approach yielded the desired results at a level of resolution comparable to that of the MiSeq method, but at a fraction of the cost. Community fingerprinting methods (especially TRFLP) remain relevant for the identification of dominant microbes in a population, the observation of shifts in plant microbiome community diversity, and for screening samples before their use in more sensitive and expensive approaches.

The development of new molecular technologies around the start of the new millennium enabled botanists to employ them to better comprehend the impressive diversity of bacteria and fungi associated with plants. Having received little attention until recently, plant microbiome research (the study of the bacteria, fungi, and other microbes inside and on the surfaces of roots, stems, leaves, flowers, and seeds) is providing many opportunities for scientists to make important biological discoveries that could, for example, help to improve agricultural productivity and plant nutrition (Johnston‐Monje et al., 2019). Like most environmental microbes, plant‐inhabiting microbes may be difficult or impossible to study using classical culture‐based techniques (Sarhan et al., 2019), thus these molecular methods have been vital for gaining a more complete understanding of their ecology (van Overbeek et al., 2006).

Molecular techniques for the study of plant‐associated microbial communities usually depend on PCR for the amplification of barcode DNA sequences corresponding to entire phylogenetic groups. In microbial ecology, the molecular targets for PCR are usually ribosomal subunit genes (rDNA), internal transcribed spacer (ITS) regions between rDNA, or protein‐coding genes that are important for specific cellular functions. The barcode DNA used for surveying bacteria is usually 16S rDNA, whereas the study of plant mycobiomes most often focuses on 18S rDNA or ITS sequences on either side of the fungal 5.8S rDNA sequence (Ranjard et al., 2001). In order to survey the broad diversity of microbial sequences present in a plant DNA extract, PCRs should be conducted using universal primers that can selectively amplify as much target microbial DNA as possible, while avoiding non‐target contaminants such as chloroplast, mitochondrial, and plant rDNA. A popular example of such universal primers is 799f paired with 1193r, which together selectively amplify bacterial 16S rDNA to the exclusion of chloroplast 16S rDNA (Thijs et al., 2017). It should be noted that while no primer pair is either universal or unbiased, some are better than others, thus primer choice has significant implications on the abundances and richness of the sequences that are amplified (Pollock et al., 2018). These amplicons may be sequenced, or separated and analyzed, using various fingerprinting approaches. Both sequence data and fingerprinting patterns can be visualized using heatmaps or histograms, and can be compared using multivariate statistics that reveal the relationships between microbial communities.

The earliest DNA‐based technique to survey plant microbiomes was to clone and separate PCR products into plasmid‐containing colonies of *Escherichia coli* and use Sanger sequencing on the resulting library to generate a list of all the microbial barcode DNA found in the environmental sample (Ward et al., 1990). While informative, the use of Sanger sequencing (costing at least US$5 each) on the hundreds or thousands of individual clones necessary to define a plant microbiome is prohibitively expensive. The development of next‐generation or high‐throughput sequencing (HTS) technologies to analyze microbial amplicons from plant DNA samples has been a significant boon to the study of plant microbiomes (Knief, 2014). HTS is advantageous because both the identification and relative DNA abundance of most (but not all) bacteria or fungi in a community can be obtained simultaneously. Examples of PCR‐dependent HTS technologies are 454 pyrosequencing (obsolete since 2016), Ion Torrent, SOLiD, Illumina, PacBio, and Nanopore. These techniques are not cheap, however; for example, an Illumina MiSeq machine (currently the most popular HTS platform for microbiome sequencing) costs over US$100,000, and each cartridge of sequencing chemicals (enough for one sequencing run) costs more than US$1000. Rather than buying your own equipment, commercial sequencing providers will charge US$2500 (or more) for one run of amplicon sequencing on an Illumina MiSeq. If one has a large number of amplicons to analyze, HTS has the advantage of allowing for sample multiplexing, which can reduce per‐sample sequencing costs. We have, for example, used barcoded sets of 24 forward and 32 reverse primers (which cost almost US$1000 to buy) to differentially label 768 different plant microbiome samples, which were pooled and sequenced on a single run of the Illumina MiSeq for US$2500, at a per‐sample cost of US$3.25. Scientists hoping to analyze just a few samples and who do not have their own HTS machine or sufficient resources to pay for multiple sequencing runs with a commercial service provider may find themselves wasting their money on excessive sequencing or sacrificing the quality of their experiment, for example, by reducing repetitions necessary for statistical confidence or analyzing incomplete/poor data sets when experimental errors arise (Prosser, 2010).

Microbial community fingerprinting techniques are an established set of molecular tools used to profile microbial communities. They include single‐strand conformation polymorphism (SSCP), amplicon length heterogeneity PCR (LH‐PCR), ribosomal intergenic spacer analysis (RISA) and automated ribosomal intergenic spacer analysis (ARISA), temperature gradient gel electrophoresis (TGGE), denaturing gradient gel electrophoresis (DGGE), and terminal restriction fragment length polymorphism (TRFLP) methods. Unlike HTS technologies, these fingerprinting techniques do not require the purchase of capital‐intensive equipment and chemicals, nor do they require expensive commercial services to function (Phadke et al., 2017). Amplicons containing diverse microbial sequences are separated using electrophoresis in acrylamide gels, producing patterns of migrating DNA bands that are then photographed, digitized, and analyzed using commercial software such as GelCompar and Bionumerics, freeware such as GelClust, or a variety of other programs reviewed by Heras et al. (2015). With a few procedural modifications, community fingerprinting methods (except DGGE) can have their fluorescently tagged amplicons analyzed on DNA‐sequencing machines for a few dollars per sample, greatly improving the resolution, reproducibility, and quality of the data. Community fingerprinting does not directly provide taxonomic information about microbes, although signals of interest can later be annotated with taxonomic information using complementary molecular or bioinformatics techniques. Molecular fingerprinting is also limited in its ability to detect rare taxa in DNA samples, and is unable to precisely estimate microbe abundance (Bent and Forney, 2008). Despite these limitations, the use of fingerprinting methods to provide rapid, inexpensive, and accurate detection of spatial, temporal, and treatment‐induced shifts in microbial communities remains widespread in plant science, as full‐scale sequencing with HTS approaches is often excessive and could be a waste of resources (Prosser, 2010). Additionally, community fingerprinting provides a rapid, relatively inexpensive option to prescreen samples for further phylogenetic analysis, for example by enabling the discovery of specific microbial signals for later taxonomic identification or screening for interesting samples that might be worth studying later with HTS (van Dorst et al., 2014). A comparison of the DNA‐based methods currently available for the study of plant microbiomes is shown in Table 1. The purpose of this review is to highlight the use of microbial community fingerprinting in plant research, paying special attention to recent (i.e., from the past 10 years) experiments that juxtapose fingerprinting data with HTS results.

**Table 1:** Comparing DNA‐based methods for studying plant microbiomes (adapted from Metzler et al., 2019).

					Relative costs			
Method	Product	Data output	Multiplexing	Unit of DNA extraction	Reagents and supplies	Analysis (96 samples)	Bioinformatics	Ability to identify microbial species
Sanger sequencing	DNA sequences (long, ~800 bp)	One sequence per clone library colony	No	Individual clone library colony	Medium (PCR cloning kits, competent cells, sequencing chemistry)	Very high (US$5 per sequence for at least 10 colonies per sample × 96 = US$4800)	None (BLASTing sequences one by one for phylogenetic annotation in GenBank)	High (each sequence maps directly onto microbial phylogeny)
SSCP/CE‐SSCP	Gel photo/DNA chromatograms	Up to one signal per conformational polymorphism (e.g., 100 signals for 100 differently structured strands)	No	Whole plant tissue or surface	Low (phosphorylated primers, lambda exonuclease)	Low (US$0 + gel photograph analysis software)	Low (statistical analysis of electrophoretic migration patterns)	Medium (bands need to be cut out, amplified, and sequenced)
DGGE and TGGE	Photo of electrophoretic gel	Up to one signal per denaturation variant (e.g., 100 signals for 100 different denaturing amplicons)	No	Whole plant tissue or surface	Medium (denaturing gradient gel electrophoresis system)	Low (US$0 + gel photograph analysis software)	Low (statistical analysis of electrophoretic migration patterns)	Medium (bands need to be cut out, amplified, and sequenced)
LH‐PCR and ARISA	DNA amplicon chromatograms	Up to one signal per size variant (e.g., 100 signals for 100 differently sized amplicons)	No	Whole plant tissue or surface	Low (fluorescently labeled primers)	Low (US$100–200 + DNA chromatogram analysis software)	Low (statistical analysis of DNA amplicon chromatograms)	Medium (bands can be annotated by size or run on gel for excision and sequencing)
TRFLP	DNA fragment chromatograms	Up to two signals per restriction site variant (e.g., 200 signals for 100 differently sized fragments)	No	Whole plant tissue or surface	Low (fluorescently labeled primers and restriction endonucleases)	Low (US$100–200 + DNA chromatogram analysis software)	Low (statistical analysis of DNA amplicon chromatograms)	Medium (bands can be annotated by size or run on gel for excision and sequencing)
High‐throughput sequencing	DNA sequences (short, ~300 bp)	Tens of thousands of sequences per sample (complete analysis of the PCR amplicon)	Yes	Whole plant tissue or surface	High (high‐fidelity Taq, platform‐specific sequencing cartridges and dozens of platform‐specific adapters for multiplexing)	High (US$2500 for one MiSeq run with a commercial provider or US$1000 for sequencing reagents on your own machine)	Medium (special training/computer programming required)	High (each sequence maps directly onto microbial phylogeny)

Note

ARISA = automated ribosomal intergenic spacer analysis; CE‐SSCP = capillary electrophoresis single‐strand conformation polymorphism; DGGE = denaturing gradient gel electrophoresis; LH‐PCR = amplicon length heterogeneity PCR; SSCP = single‐strand conformation polymorphism; TGGE = temperature gradient gel electrophoresis; TRFLP = terminal restriction fragment length polymorphism.

## Single‐strand conformational polymorphism (SSCP)

SSCP was developed in the 1980s as a method of detecting polymorphisms and mutations in human DNA by comparing chemically denatured PCR product migration patterns through electrophoresis gels (Schmalenberger and Tebbe, 2014). In modern SSCP, target amplicons are generated by performing PCRs with universal primers, one of which is 5′‐end phosphorylated. The amplicons are then heat denatured and digested with lambda exonuclease, destroying the phosphorylated single‐strand DNA prior to electrophoresis in non‐denaturing acrylamide gels. The nucleotide sequence of the single‐strand DNA dictates its 3D structure, which in turn alters its electrophoretic mobility in unpredictable ways; changes in a single nucleotide can have a very strong impact, with substitutions of a single base altering the migration pattern of a 300‐base sequence in one study (Schmalenberger and Tebbe, 2014). It is very important to maintain a constant temperature during electrophoresis, as gels run with the same samples at different temperatures will appear completely different, making it difficult to obtain reproducible results. If you just want to compare community fingerprints from different samples, taking a gel photo and analyzing the patterns is your last step; however, at this stage it is also possible to identify the taxonomy of interesting bands by cutting them out, reamplifying, and sequencing them. Alternatively (and for the price of a few dollars per sample), the capillary electrophoresis variant of SSCP (CE‐SSCP) includes a fluorescently labeled PCR primer and runs the denatured amplicons through a sequencer for the automated detection of fluorescent strand sizes.

The majority of papers referencing the use of SSCP to profile plant‐associated microbial communities focus on rhizosphere populations of bacteria. For example, using SSCP of bacterial 16S rDNA, soil salinity has been shown to increase the diversity of bacteria in sugarcane (*Saccharum officinarum* L.) rhizospheres (Lamizadeh et al., 2019), whereas maize (*Zea mays* L.) rhizobacterial populations have been shown to significantly vary by soil salinity levels, organic carbon, calcium, and geography (Castellanos et al., 2009). SSCP was used to profile bacterial populations in healthy and necrotic wood tissues of mature Tunisian grapevines (*Vitis vinifera* L.), aiding in the discovery and isolation of 19 unique endophytes with biocontrol potential (Rezgui et al., 2016). Similarly, in an intriguing methods paper, Zachow et al. (2013) use SSCP to monitor bacterial populations in bait plants, including maize, oilseed rape (*Brassica napus* L.), sorghum (*Sorghum bicolor* (L.) Moench), and sugar beet (*Beta vulgaris* L.), which are intended to capture agriculturally useful microbes from stress‐resistant alpine mosses, lichens, and primrose (*Primula vulgaris* Huds.). SSCP was used to show that these bait plants absorb, on average, 10% of their microbiome from the stress‐resistant inoculum, providing targets for bioprospecting and the development of plant probiotics (Zachow et al., 2013).

A comparison of SSCP and HTS suggests that SSCP is not always sufficiently sensitive to detect differences between bacterial populations. The SSCP of 16S rDNA was contrasted with 454 pyrosequencing in the study of the influence of six different bacterial inoculants on the bacteriomes of Egyptian‐grown chamomile plants (*Chamomilla recutita* (L.) Rauschert); while the community fingerprinting method did not suggest any treatment effects, the greater sensitivity of pyrosequencing detected a clear shift within the community structure and the corresponding beta diversity indices (Schmidt et al., 2014). Both methods suggest a remarkable stability of lettuce (*Lactuca sativa* L.) phyllosphere bacterial populations in response to slug and snail herbivory, although 454 sequencing revealed alterations in the abundance of *Escherichia* and *Enterobacter* DNA after gastropod feeding (Erlacher et al., 2015). Other studies using both SSCP and 454 sequencing to study bacterial populations in plants yield congruent results; for example, rhizosphere bacterial populations of wild beet ancestors (*Beta vulgaris* subsp. *maritima* (L.) Arcang.) were revealed to be significantly different and more diverse than those of modern sugar beet using both methods (Zachow et al., 2014). Likewise, both methods show that chamomile, pot marigold (*Calendula officinalis* L.), and *Solanum distichum* Schumach. & Thonn. planted at a desert farm in Egypt were able to alter 60% of the bacterial diversity in bulk soil around their roots, whereas 20% of endophytes and rhizobacteria were shared among plants (Köberl et al., 2011). Neither SSCP nor Illumina MiSeq were able to detect the effect of *Xanthomonas* wilt resistance transgenes (*hrap* and *pflp* from sweet pepper [*Capsicum annuum* L.]) on bacterial populations in rhizospheres or endospheres of transgenic bananas (*Musa acuminata* Colla) growing in Ugandan fields (Nimusiima et al., 2015).

SSCP has also been used to study plant‐associated fungal populations. For example, SSCP of arbuscular mycorrhizae 18S rDNA showed that 19 years after the addition of urban refuse to highly eroded, semiarid soils in Spain, there had been a significant increase in the diversity of *Glomus* fungi around the roots of *Phagnalon rupestre* (L.) DC., *Piptatherum miliaceum* (L.) Coss, *Stipa parviflora* Desf., and *Plantago lagopus* L. (del Mar Alguacil et al., 2009). Using CE‐SSCP to study the fungi involved in grapevine trunk disease, it was possible to observe seasonal fluctuations in endophyte populations within woody tissues, but not between mycobiomes of symptomatic and asymptomatic vines (Bruez et al., 2014). A study of fungal epiphyte diversity of beech trees (*Fagus sylvatica* L.) showed that CE‐SSCP was as effective at detecting inter‐tree variation as 454 sequencing (Cordier et al., 2012). The relatively affordable nature of CE‐SSCP enabled the researchers to process more leaf samples (243 leaves from nine trees) than with the more expensive HTS technique, leading to the conclusion that most of the variance in fungal epiphyte populations exists between leaves of the same group, rather than between different branches or different trees. In other words, analyses of the larger numbers of samples and repetitions made possible using inexpensive techniques can lead to more nuanced research findings.

## Denaturing gradient gel electrophoresis (DGGE) and temperature gradient gel electrophoresis (TGGE)

The use of DGGE with rDNA for the analysis of microbial communities was first described in 1992 in a study of bacterial biofilms from wastewater (Muyzer and Smalla, 1998). The technique allows DNA fragments of the same length (up to 500 bp) to be separated during electrophoresis through polyacrylamide gels containing a linear gradient of DNA denaturants, usually a mixture of urea and formamide. As the DNA migrates though the gel, it is subjected to increasingly extreme denaturing conditions until reaching its denaturing threshold, where it transitions from a helical to a partially melted molecule, practically halting its migration through the gel (Muyzer and Smalla, 1998). Sequence variation within amplified DNA molecules causes their denaturing thresholds to differ, meaning that taxonomically distinct amplicons stop migrating at different positions in the gel. DGGE allows an average of 50% of sequence variants to be observed; however, by using PCR primers that attach a 30–50‐bp GC‐rich sequence to the amplicons, blocking the complete denaturation of dsDNA, nearly 100% of sequence variants can be detected (Muyzer and Smalla, 1998).

One technical limitation of DGGE is the ability to cast gels with consistent and reproducible gradients of denaturant, which make it very difficult to reproduce results during different experiments. For DGGE gel casting, there is expensive commercial equipment available, as well as cheaper low‐tech options (Yang et al., 2010). Rather than try to vary the chemical gradient, an alternative is to create a temperature gradient. In TGGE, the amplicon of interest is loaded into a polyacrylamide gel with a constant concentration of denaturants, but as the DNA migrates during electrophoresis, the temperature of the whole system is increased gradually, resulting in a linear temperature gradient over the duration of the run. As different sequences reach their temperature denaturation threshold, they partially melt and their migration stops. Bands in DGGE/TGGE gels can be visualized by staining them with DNA dyes such as ethidium bromide, GelRed, or SYBR Safe, and can then be photographed and digitized for the further analysis of community profiles. As in SSCP, bands of interest can be cut out of the gel and taxonomic information about the microbe elucidated by subsequent sequencing. Neither DGGE nor TGGE is compatible with capillary electrophoresis, which limits the accuracy and throughput of these techniques in comparison with CE‐SSCP, for example. Another major limitation of DGGE/TGGE is the consistency from run to run and user to user, with poor reproducibility making it difficult to replicate results or to annotate bands without cutting out individual bands and sequencing them.

DGGE of 16S rDNA has been a popular choice for profiling bacterial populations in rhizospheres, endospheres, and phyllospheres. DGGE and band sequencing amplified from surface‐sterilized endospheres of the Chinese orchid *Dendrobium officinale* Kimura & Migo resulted in 29 dominant bands that were mostly of the genus *Burkholderia* (Yu et al., 2013). In all eight sampled phyllospheres of the Antarctic grass *Deschampsia antarctica* E. Desv., DGGE revealed four common bands representing *Agrobacterium*,* Aurantimonas*,* Pseudomonas*, and *Psychrobacter* (Cid et al., 2017). For the Mediterranean seagrass *Posidonia oceanica* Delile sampled from 26 locations around the Balearic Islands, DGGE revealed patterns composed of 34 different bands that were differently distributed among tissues and sampling locations (Garcias‐Bonet et al., 2012). A pioneering study used DGGE to show that 45% of bacterial endophytes of rice (*Oryza sativa* L.) are transmitted from generation to generation through seeds, but that external soil conditions (especially pH) alter the endophyte community composition of the growing plant (Hardoim et al., 2012). Other DGGE studies of 16S rDNA have helped show that the invasive grass *Sorghum halepense* Pers. recruits bacterial endophytes from the soil as well as its own rhizomes (Rout et al., 2013), while peppers grown under drought stress are able to alter and enrich their rhizobacterial populations to enhance plant photosynthetic activity and biomass accumulation (Marasco et al., 2012). Furthermore, DGGE was used to show that the inoculation of domesticated grapes with beneficial endophyte strains from wild grapes is able to dramatically alter the structure of the rhizosphere bacterial community while increasing shoot, root, and leaf biomass (Rolli et al., 2015). Intriguingly, DGGE has also been used to discover cultivation‐resistant endophytes. Tissue‐cultured seedlings germinated from seeds of *Ziziphus jujuba* Mill. var. *fupingdazao* contained no cultivatable bacteria, but microscopic analysis showed there to be spherical and rod‐shaped microbes present, and DGGE was subsequently used to reveal at least six dominant bands representing seed‐transmitted endophytes (Yang et al., 2010). Similarly, after five years of sterile in vitro tissue culturing of pineapple (*Ananas comosus* (L.) Merr.) plants, a DGGE of bacterial 16S rDNA revealed three separate bands in roots and leaves that were sequenced and identified to be endophytic *Burkholderia*,* Pseudoxanthomonas*, and *Stenotrophomonas* (Abreu‐Tarazi et al., 2010).

DGGE has also been used to study plant‐associated mycobiomes, although few examples have been published in the past 10 years. DGGE of fungal 18S rDNA has been used to determine whether *Trichoderma harzianum* SQR‐T037 could “bio‐fumigate” cucumber (*Cucumis sativus* L.) rhizosphere soil in order to control Fusarium wilt (Chen et al., 2012), and to observe that the fungal biocontrol *Pseudomonas fluorescens* 2P24 transiently alters fungal populations in cucumber rhizospheres after inoculation (Gao et al., 2012). This technique has also been used to generate 40 different bands from the families Dothideomycetes, Leotiomycetes, Lecanoromycetes, and Sordariomycetes that were associated with fresh or decaying pine needles from *Keteleeria fortunei* Carrière, *Pinus elliottii* Engelm., and *Pinus massoniana* Lamb. (Jeewon et al., 2018). A study of fungi colonizing *Phytolacca americana* L., *Rehmannia glutinosa* Steud., *Perilla frutescens* (L.) Britton, *Litsea cubeba* Pers., and *Dysphania ambrosioides* L. growing in heavy‐metal‐polluted soil near the Chinese Dabaoshan mine used DGGE to identify many species of arbuscular mycorrhizae, especially of the genus *Glomus*, which predominated in all root samples (Long et al., 2010).

As one of the more popular techniques for fingerprinting plant‐associated bacterial populations, there are many instances where investigators have used the “classic” method of DGGE alongside “modern” HTS techniques such as 454 pyrosequencing (an early HTS platform). Usually, this juxtaposition of techniques is used as a way to cross‐validate methodologies, although in some cases it has been employed to economically prescreen samples before using the more sensitive and expensive HTS. DGGE and 454 pyrosequencing yielded similar information on bacterial population diversity in a study of the effect of transplantation on mangrove rhizobacterial populations (Cleary et al., 2012), as well as in an analysis of rhizobacterial communities of Chilean plants growing in extreme environments including the Atacama Desert, the Andes Mountains, and the Antarctic (Jorquera et al., 2016). Similar results were also obtained for both methods in an evaluation of the impact that soil type has on bacterial diversity in lettuce rhizospheres (Schreiter et al., 2014), an investigation of the differences among the bacteria living in the phyllospheres of different *Arabidopsis thaliana* (L.) Heynh. cuticular wax mutants (Reisberg et al., 2013), and a characterization of microbiome transmission from the nematode causing pine wilt disease to its insect vector and diseased pine trees (Alves et al., 2018). Bacterial populations have also been simultaneously characterized using DGGE and Illumina MiSeq, for example, in the observation of wheat (*Triticum aestivum* L.) microbiome transmission during germination and growth on sterile vermiculite (Qin et al., 2016), as well as in the study of endophytes in *Dendrobium officinale*, where culturing identified seven genera, DGGE identified 16, and MiSeq identified 449 (Pei et al., 2017). In the later publication, the authors concluded that “DGGE is an alternative to investigating primary diversity patterns; however, the metagenome method is still the best choice for determining the endophytic profile in plants” (Pei et al., 2017). Rather than compare methods, DGGE was used in a prescreen to select high‐quality, low‐variation cactus (*Myrtillocactus geometrizans* Console and *Opuntia robusta* H. L. Wendl. ex Pfeiff.) DNA samples for later sequencing on the more expensive Illumina MiSeq platform (Fonseca‐García et al., 2016).

## Amplicon length heterogeneity PCR (LH‐PCR) and automated ribosomal intergenic spacer analysis (ARISA)

Across evolutionary time, genetic loci tend to accumulate insertion or deletion mutations that result in length polymorphisms—differences that can be correlated to phylogeny and used to create community fingerprints to profile microbial diversity in environmental DNA. LH‐PCR was first used in microbial ecology in 1998, when it was applied to estimate the community composition of bacterioplankton in ocean water by analyzing the variation of rDNA size (Suzuki et al., 1998). Although LH‐PCR products can be separated using polyacrylamide gel electrophoresis (as in SSCP or DGGE), the resolution, reproducibility, and analysis time is much improved by instead using fluorophore‐tagged primers to create fluorescent amplicons that are analyzed using capillary electrophoresis and laser detection in DNA sequencing machines. This technique has also been called fluorescent amplified fragment length polymorphism, and is used for identifying plant roots in soil samples (Metzler et al., 2019). The most common variant of LH‐PCR for studying microbiomes is ARISA. ARISA community fingerprints are electropherograms reflecting the highly diverse sizes of the ITS region between 16S rDNA and 23S rDNA in bacteria, or the ITS region between the 18S and 28S rDNA in fungi (Ranjard et al., 2001). ARISA is a good choice for community fingerprinting, as the ITS regions of bacteria and fungi have greater variability in both sequence composition and length than the coding regions of genes such as rDNA. Both LH‐PCR and ARISA have useful aspects, in that they provide phylogenetically relevant fragment length data that can be directly associated with specific taxonomic sequences archived in databases, and they both save time and/or money by requiring no additional processing after PCR. Because LH‐PCR is only useful in surveying naturally occurring diversity in DNA amplicon length, great care must be taken to select hypervariable loci that will allow different microbes in the population to be observed.

There are few examples of recent studies using LH‐PCR to study plant microbial populations, probably because the bioinformatically optimal DNA targets (i.e., bacterial 16S rDNA) do not vary dramatically in size from species to species. Nevertheless, LH‐PCR targeting the bacterial 16S rDNA has been a quick and economical method for screening endophytic communities of a large number of phytoplasma‐infected grape leaves (Bulgari et al., 2014), to distinguish between the rhizospheres of switchgrass (*Panicum virgatum* L.) and jatropha (*Jatropha curcas* L.) (Chaudhary et al., 2012), and to estimate the diversity of endophytic bacteria in the medicinal plants *Codonopsis pilosula* Nannf., *Ephedra sinica* Stapf, and *Lamiophlomis rotata* Kudô (Li et al., 2013). LH‐PCR of rDNA was also used to investigate whether subboreal forest soil contamination with petroleum alters the spatial patterns of ectomycorrhizal and ericoid mycorrhizal fungal in the shared rhizosphere of *Pinus contorta* var. *latifolia* Engelm. ex S. Watson and lingonberry (*Vaccinium vitis‐idaea* L.) (Robertson et al., 2011). Rather than focus on rDNA, LH‐PCR and TRFLP based on variation in the *nifH* gene has been used to investigate the response of nitrogen‐fixing bacteria in Jerusalem artichoke (*Helianthus tuberosus* L.) rhizospheres to different amounts of nitrogen fertilizer (Meng et al., 2012). Although both methods showed that the supply of nitrogen fertilizer negatively influenced diazotroph DNA abundance, TRFLP detected a greater diversity of diazotrophs than did LH‐PCR.

With a greater heterogeneity in ITS sequence length than that of rDNA, RISA is a better choice for studying plant microbiology than is LH‐PCR. Bacterial RISA has been used to help find novel copper‐tolerant bacterial endophytes inside *Haumaniastrum katangense* L. and *Crepidorhopalon tenuis* (S. Moore) Eb. Fisch. plants growing in the Katangan Copper Belt, with amplicons being screened in polyacrylamide gels and distinct bands sequenced (Cubaka Kabagale et al., 2010). Another study used RISA to show that transgenic *Brassica napus* carrying *NicC* (chitinase with antifungal activity) does not have an altered microbial rhizosphere community (Khan et al., 2017), whereas ARISA of bacterial populations in 12 alpine plant species showed that each plant species selects for a different rhizobacterial community (Ciccazzo et al., 2014). Bacterial ARISA (in combination with clone library sequencing) has been used as a quick and economical way to monitor the efficiency of bacterial endophyte enrichment protocols (Ikeda et al., 2009). A recent doctoral thesis studying the mycobiomes of *Fraxinus excelsior* L., *F. ornus* L., and *Acer pseudoplatanus* L. leaves employed fungal ARISA to cheaply monitor the results of PCR methods using novel peptide nucleic acid blockers targeting plant rDNA (Schlegel, 2018). Yet another method paper developed a new ARISA protocol for fungi and evaluated its utility in distinguishing between endophytes in twigs and leaves of a variety of tree species including *Acer platanoides* L., *A. pseudoplatanus*,* Betula pendula* Roth, *Fagus sylvatica* L., *Picea abies* (L.) H. Karst, *Pinus sylvestris* L., *Quercus petraea* (Matt.) Liebl., and *Q. robur* L. (Weig et al., 2013). Based on this ability to quickly, clearly, and economically distinguish differences in fungal population diversity, the authors suggest that their improved ARISA protocol is “particularly useful for environmental screening and monitoring projects.”

Few studies report the use of ARISA in tandem with HTS methods. In one study, 454 pyrosequencing was used to evaluate the sensitivity and accuracy of ARISA fingerprints, with the relationships between fungal communities associated with four salt‐marsh plants found to be consistent between both methods, supporting the idea that ARISA may be used to provide a quick snapshot of diversity before further refining microbiome taxonomic data using HTS methods (Gillevet et al., 2009). A similar confirmation of congruency between ARISA and the pyrosequencing of microbial communities was achieved in a study of the impacts of nitrate fertilization on rhizobacterial communities in nitrate‐signaling mutants of *Arabidopsis thaliana* (Konishi et al., 2017), as well as in a paper attempting to link differences in *Medicago truncatula* Gaertn. genotypes and phenotypes to changes in rhizobacterial community structure (Zancarini et al., 2013), and in attempts to better understand the microbiology of apple replant disease by profiling bacteria and fungi in soils before and after planting (Franke‐Whittle et al., 2015).

## Terminal restriction fragment length polymorphisms (TRFLP)

Conserved DNA, such as the coding regions of genes (i.e., bacterial 16S or fungal 18S rDNA), tends to accumulate fewer insertion or deletion mutations over evolutionary time than do non‐coding regions (i.e., ITS sequences), greatly limiting the use of genetic locus size for distinguishing between strains, species, genera, and even families of bacteria or fungi. In these cases, techniques that compare the DNA sequence, rather than its length, can prove advantageous. SSCP and DGGE allow similarly sized DNA sequences to be distinguishing by contrasting their sequence‐determined conformation or denaturing thresholds, whereas restriction fragment length polymorphism (RFLP) analyses sample DNA sequence diversity with restriction endonucleases. Amplicons of barcode DNA from microbial communities are cut into diagnostic fragment patterns when separated by size on an electrophoretic gel. When RFLP is used to genotype individual microbes by the amplification of their ribosomal DNA combined with restriction analysis, it is called ARDRA. In contrast, when the amplicon (from either single strains or entire microbial communities) is produced with fluorescently labeled primers that are cut by restriction enzymes and visualized in electropherograms, the technique is called TRFLP. As the name suggests, TRFLP only collects size and signal intensity data on the two fluorescently labeled “terminal fragments” resulting from digestion with a restriction endonuclease. Because enzyme selection is very important for maximizing terminal fragment diversity (which is intended to correlate with phylogenetically different members of the microbial community), it has already been optimized for common TRFLP targets such as bacterial 16S rDNA (Schütte et al., 2008) and fungal ITS DNA (Alvarado and Manjón, 2009). TRFLP fingerprints can be analyzed with multivariate statistics to compare microbial populations, but individual terminal restriction fragment (TRF) sizes are also useful for taxonomic prediction by comparison to sequence databases (either publicly available or resulting from clone library sequencing) using tools such as MiCA (Shyu et al., 2007), TRiFLe (Junier et al., 2008), or even Restriction Mapper (http://www.restrictionmapper.org). To directly identify TRFs, it is also possible to replace the primer's fluorescent tag with biotin, purify the TRFs using streptavidin‐coated magnetic beads, run the TRFs on an agarose gel, and then cut out and sequence them using a protocol called physical capture TRFLP (Blackwood and Buyer, 2007).

With advantages of speed, high resolution, and high reproducibility, it is not surprising that TRFLP is the most popular community fingerprinting method among plant biologists. It continues to be used to profile plant microbiomes and to elucidate which experimental or environmental factors influence microbial community structure and diversity. Recent examples of its use include an exploration of how rice genotype and seed transmission influence plant endophyte populations (Walitang et al., 2018, 2019), and in a study showing that foliar application of plant‐derived bioactive compounds alter lettuce leaf bacterial populations of *Pantoea*,* Pseudomonas*,* Acinetobacter*, and *Bacillus* (Luziatelli et al., 2019). It was also used to reveal that inoculation of two *Methylobacterium* spp. endophytes onto potato (*Solanum tuberosum* L.) and pine (*Pinus sylvestris* L.) improves plant resistance to pathogens and shifts the structure of the bacterial endophyte community (Ardanov et al., 2012), and to show that the biocontrol soil inoculant *Bacillus amyloliquefaciens* temporarily changed the structure and diversity of tomato (*Solanum lycopersicum* L.) rhizosphere bacteria and reduced the symptoms of Fusarium wilt (Wan et al., 2018). TRFLP was used to profile the bacterial endophyte communities associated with the leaves of the most agriculturally important genotypes of mature rice plants in Uruguay, revealing that although each variety had distinct populations of bacterial endophytes, 74% of their diversity was shared (Ferrando et al., 2012). TRLFP has also been useful to show that the bacterial and diazotroph communities residing in the endophytic compartment of the bioenergy crop *Miscanthus ×giganteus* J. M. Greef & Deuter ex Hodk. & Renvoize do not vary from region to region or soil to soil, while rhizosphere communities do (Li et al., 2016).

**Figure 1:**
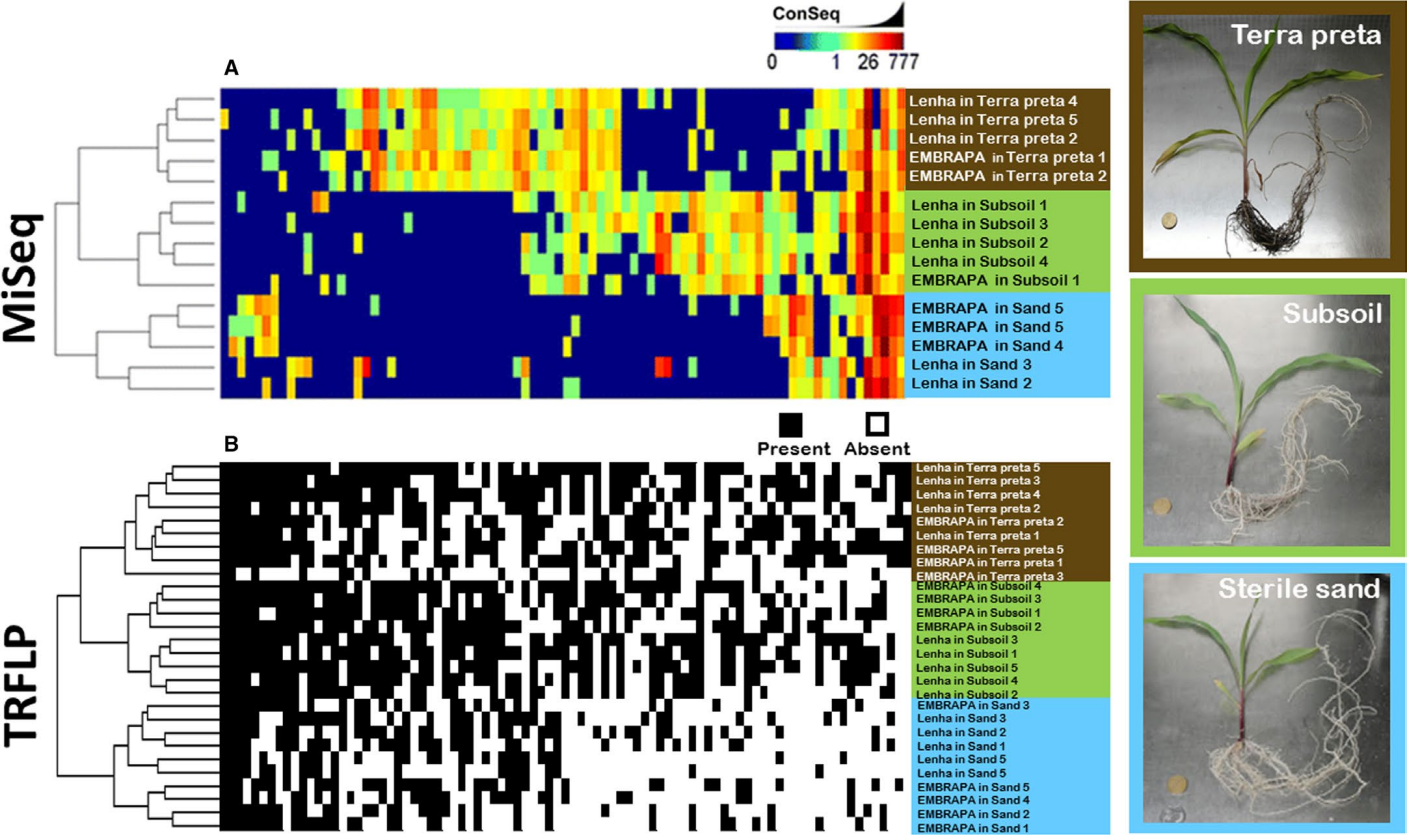
Profiling and comparing rhizosphere populations of bacteria from two Brazilian maize genotypes growing on three different substrates: sterile sand (blue), subsoil (grey), and terra preta do indio (brown). The same rhizosphere DNA samples were analyzed to perform (A) MiSeq, using primers 515F and 806R, and (B) TRFLP, using primers 27 F‐Degen and 1492r, followed by a nested PCR with fluorescent primers 799f and 1389r, then restriction using the *Dde*I enzyme. The MiSeq data are displayed as a heatmap of read copy numbers (from 0–777) observed in each sample, while TRFs are displayed as present (black) or absent (white). The clustering of rhizosphere profiles was accomplished using a Bray‐Curtis dissimilarity of log_2_‐transformed read numbers (MiSeq) or TRF presence/absence. Adapted from Johnston‐Monje et al. (2016).

Over the past decade, we have used TRFLP to study the patterns of co‐evolution between maize genotypes and seed endophyte populations (Johnston‐Monje and Raizada, 2011), leading to the startling observation that the majority of maize bacterial endophytes derive from the seed rather than the surrounding soil (Johnston‐Monje et al., 2014). TRFLP was also used alongside Illumina MiSeq to establish the provenance of rhizosphere bacteria in maize, with results published a few years ago (Johnston‐Monje et al., 2016). In that study, a modern Brazilian commercial maize genotype was compared with an unimproved landrace called Lenha; both were grown on sterile sand, a powdery subsoil from 400 m underground, and terra preta do indio, a biochar from the Amazon jungle. Both TRFLP and Illumina MiSeq methods were used to characterize the bacterial communities in maize rhizospheres, and Bray Curtis dissimilarity clustering was performed to ordinate the binary (TRFLP) or log_2_ (MiSeq) transformed data (Fig. 1). The ordination of data derived from both methods produced identical clusters of rhizobacterial communities, which grouped by substrate first and then, to some extent, by genotype. Both approaches also revealed shared bacterial signals across sample types, which was interpreted to mean that a significant proportion of the maize rhizosphere comes from the seed and not the soil. Although TRFLP and MiSeq allowed the same conclusions to be made about the impact of soil on maize rhizosphere microbiology, TRFLP had the clear cost advantage over MiSeq, costing about US$75 to run the 30 samples on a sequencing machine at a Canadian university (at academic prices) versus more than US$1000 to run 24 pooled samples on a MiSeq machine that cost US$125,000 to buy.

Although the study of mycobiomes lags behind that of bacteriomes, TRFLP is also being used to study plant‐associated fungi. It has been used to show that epiphytic fungal richness on leaves of *Quercus ilex* L. increases during the dry season, while endophyte richness decreases (Penuelas et al., 2012), and was also used to estimate the richness, composition, and abundance of fungal endophyte DNA in the leaves of apple (*Malus domestica* (Suckow) Borkh.) cultivars and reveal a correlation between richness and resistance to the pathogen *Alternaria mali* (Hirakue and Sugiyama, 2018). Furthermore, TRFLP was used to evaluate the impact of the *Striga* bioherbicide *Fusarium oxysporum* f. sp. *strigae* strain “Foxy‐2” on Kenyan maize mycobiomes (Zimmermann et al., 2016), and was used to prescreen DNA samples of *Brachypodium distachyon* (L.) P. Beauv. rhizospheres before sending them for further analysis on the Illumina MiSeq platform (Kawasaki et al., 2016). In a large study of grassland soil microbial communities, the authors used TRFLP to monitor microbiomes, rationalizing: “This low‐cost high‐throughput method can perform as well as deep sequencing when investigating ecological patterns in microbial communities at local to regional scales and provides qualitatively similar data for modeling community dynamics” (Sayer et al., 2017). By analyzing the TRFLP profiles of soil microbes from 78 geographically separate sites, they found that shifts in soil microbial community structure were most influenced by environmentally controlled modifications of plant physiology that provide resources to soil microorganisms (e.g., rhizo‐exudation).

**Figure 2:**
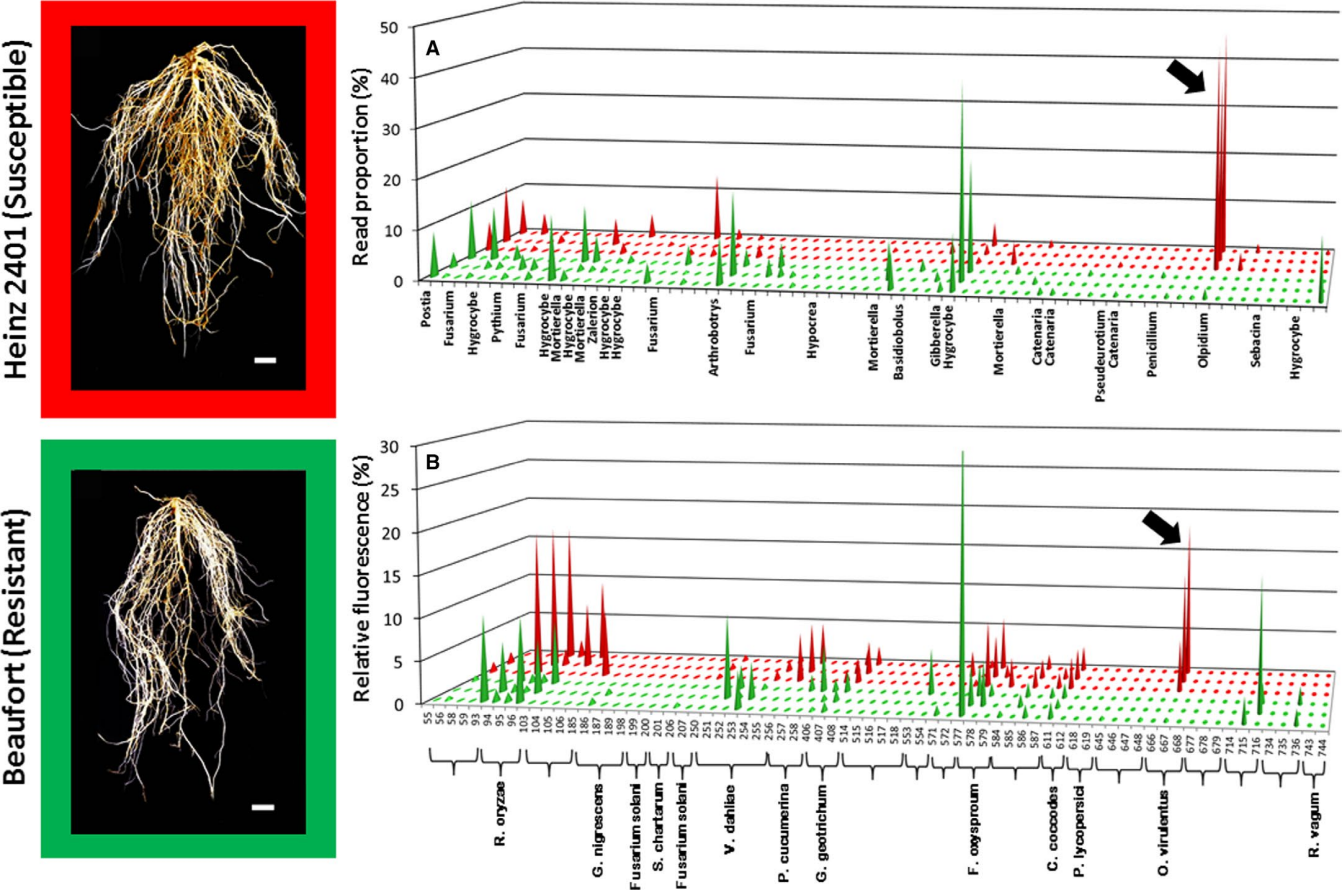
Comparing methods of acquiring mycobiome data from the endospheres of tomato vine decline (TVD)‐susceptible (in red) or ‐resistant (in green) roots. DNA from three root samples each of Heinz 2401 (susceptible) and Beaufort (resistant) plants grown in a TVD‐infested soil was amplified using fungal primers ITS‐1F and ITS‐2 and sent for MiSeq analysis, while TRFLP data were obtained after amplification with fluorescently labeled ITS‐1F and ITS‐4, followed by restriction with the *Bst* NI enzyme. The MiSeq data (A) is shown as the proportion of reads per sample represented by each OTU, while the TRFLP data (B) is the proportion of each ITS‐4 terminal fragment fluorescence as a percentage of each sample's total fluorescence. Where available, the OTUs and TR fragments are annotated with genus or species information. Scale bars = 10 mm. Large black arrows point to signals representing *Olpidium virulentus*, the suspected causal agent of TVD. TRFLP data adapted from Johnston‐Monje et al. (2017).

Our recent publication searching for the cause of an emerging tomato disease in Canada is another good example of using TRFLP to study plant mycobiomes (Johnston‐Monje et al., 2017). The published study revolved around a TRFLP‐based survey to search for fungi preferentially infecting susceptible tomato roots growing in disease‐infested farm soil. Fungal ITS‐1F/ITS‐4 amplicons were cut with the restriction enzyme *Bst*NI, with the resulting ITS‐4 fragments in the size range of 666–668 bp accounting for an average relative fluorescence of 13% per sample, whereas in resistant roots they were barely detectable at 0.2% (Fig. 2B). This diagnostic pattern drew attention to this fragment, which was then identified by physical capture TRFLP (Blackwood and Buyer, 2007) and found to represent the obligate biotrophic pathogen *Olpidium virulentus*.

To validate the TRFLP results using a more modern method, new data presented here (Fig. 2A) show the results of analyzing these same six root DNA samples on the Illumina MiSeq platform. The DNA was subjected to a 35‐cycle PCR amplification with fungal ITS‐1F/ITS‐2 primer pairs containing error‐correcting 12‐bp barcoded primers and 25‐bp adapters. PCR products were quantified using the PicoGreen assay (Thermo Fisher Scientific, Waltham, Massachusetts, USA), pooled in equimolar concentrations, and cleaned using the MO BIO UltraClean kit (QIAGEN, Hilden, Germany). Cleaned DNA pools were sequenced on an Illumina MiSeq instrument (Illumina, San Diego, California, USA) and reads were then demultiplexed, quality‐filtered, merged, and clustered into operational taxonomic units (OTUs) following the default UPARSE pipeline (Edgar, 2013). OTUs were provided taxonomic classifications using the RDP classifier (available at https://rdp.cme.msu.edu/classifier) trained on the RDP Warcup training set version 2 database. OTUs that were classified as archaea or not assigned taxonomic classifications with at least phylum‐level specificity were removed because they were likely to be technical artifacts. To then compensate for statistical differences caused by variation in read number, each sample was rarefied to include only 1000 reads. This HTS approach identified only one major OTU displaying the same diagnostic DNA abundance in susceptible roots that was observed with TRFLP, making up an impressive 43% of the reads (on average) versus only 0.7% in resistant roots (Fig. 2A). The OTU was found to share 100% identity with *Olpidium virulentus* and was by far the most abundant fungus observed inside susceptible tomato roots. Although both methods annotated a surprisingly small number of fungi (MiSeq:29 TRFLP:14) and identified *O. virulentus* as the possible cause of disease, neither TRFLP nor MiSeq was able to annotate all their data with taxonomic information (many Illumina‐derived OTUs mapped onto “unknown fungal clone”), leaving a high number of unidentified microbes in these tomato root samples. Another important lesson to be gleaned from this juxtaposition is that the picture of microbial diversity present in tomato roots seems to be influenced by the method used to observe it. For example, several abundant OTUs were identified by Illumina sequencing as belonging to the genus *Hygrocybe*, although no TRFLP signal was annotated as such. The contrast in observed microbial diversity between samples might be explained by primer bias (De Beeck et al., 2014), as TRFLP depended on amplicons derived from fluorescently labeled ITS‐1F/ITS‐4, whereas Illumina sequencing used ITS‐1F/ITS‐2 primers incorporating 12‐bp‐long indexes and 25‐bp‐long adapters. Another possible reason for the taxonomic disparity might be the result of unequal phylogenetic resolution caused by differences in amplicon sequence length; MiSeq‐derived OTUs were only 185 bp long, whereas cloned sequences from TRFLP were up to 800 bp in length. Despite their differences, both methods show the same striking pattern of *Olpidium* DNA abundance within susceptible tomato roots and its near absence in resistant roots (Fig. 2). The TRFLP approach costs much less than MiSeq, however (about US$2500 for an entire run with a commercial service provider in the United States), requiring only about US$25 for analysis on a university sequencer, plus small additional costs for the biotinylated primer (US$50) and streptavidin‐coated magnetic beads (US$100–200) needed for physical capture PCR (Blackwood and Buyer, 2007).

## CONCLUSIONS

As botanists around the world unravel the dynamics and functions of plant‐associated microbial populations, community fingerprinting continues to be an important method in their microbial ecology toolbox. Because fingerprinting is less expensive than HTS, it is possible to perform greater numbers of repetitions or prescreen samples before using more expensive techniques, which can benefit an experiment's statistical robustness and data quality (Prosser, 2010). The numerous studies reviewed here provide evidence that fingerprinting methods continue to be useful for detecting significant spatial, temporal, and treatment shifts in plant‐associated microbial communities, making full‐scale HTS an excessive (and unnecessarily expensive) option if the main experimental goals can be met without it. Plant scientists are also using community fingerprinting techniques to help them prescreen samples before subjecting them to more sensitive or costly methods of analysis. In some labs and countries where access to the resources and facilities for HTS are not readily available, these techniques are still the best methods available for plant microbiome studies. The cheapest version of these techniques (not available for either ARISA or TRFLP) would involve the running of samples on polyacrylamide gels, employing photography and digital transformation to generate DNA fingerprints; however, the high reproducibility and quality of data in electropherograms emitted by DNA sequencers more than justifies the extra expense of a few dollars per sample. When the experiment requires taxonomic information about the plant's microbiome, all community fingerprinting methods have options available for identification of a particular signal, although for TRFLP, band excision and sequencing have to be conducted separately from electronic detection. DGGE and TRFLP are the most popular community fingerprinting methods for studying plant microbiomes, with numerous publications juxtapositioning their use alongside pyrosequencing or Illumina MiSeq as a way to cross‐validate results, or as a method of flexibly and economically prescreening samples before more expensive HTS methods are used. As the first author's experience using TRFLP also shows, affordable community fingerprinting techniques can allow for both the identification of specific players in plant–microbe interactions (i.e., discovering a tomato pathogen) and the elucidation of novel biological principles (e.g., the majority of maize endophytes come from its seeds), proving that it is not necessary to have very deep pockets to make important plant‐microbiome‐related discoveries.

## AUTHOR CONTRIBUTIONS

D.J.M. and J.L.M. both contributed to the writing of this manuscript. D.J.M. prepared all figures and tables.
